# Abnormal intra-network architecture in extra-striate cortices in amblyopia: a resting state fMRI study

**DOI:** 10.1186/s40662-019-0145-2

**Published:** 2019-07-09

**Authors:** Zhuo Lu, Yufeng Huang, Qilin Lu, Lixia Feng, Benedictor Alexander Nguchu, Yanming Wang, Huijuan Wang, Geng Li, Yifeng Zhou, Bensheng Qiu, Jiawei Zhou, Xiaoxiao Wang

**Affiliations:** 1 Center for Biomedical Engineering, University of Science and Technology of China, Hefei, Anhui 230027 People’s Republic of China; 2 Hefei National Laboratory for Physical Sciences at the Microscale and School of Life Science, University of Science and Technology of China, Hefei, Anhui 230027 People’s Republic of China; 3 CAS Key Laboratory of Brain Function and Diseases and School of Life Sciences, University of Science and Technology of China, Hefei, Anhui 230027 People’s Republic of China; 4 Shenyang Aerospace University, Shenyang, Liaoning 110136 People’s Republic of China; 5 Department of Ophthalmology, First Affiliated Hospital of Anhui Medical University, Hefei, Anhui People’s Republic of China; 6 Asia Pediatric Ophthalmologist Association, Rm 2006, CC Wu Bldg., 302-308 Hennessy Rd., Wanchai, Hong Kong, People’s Republic of China; 7 School of Ophthalmology and Optometry and Eye hospital, Wenzhou Medical University, Wenzhou, Zhejiang, 325003 People’s Republic of China

**Keywords:** Amblyopia, fMRI, Resting state, Extra-striate cortices, Visuospatial network, Graph analysis

## Abstract

**Background:**

Amblyopia (lazy eye) is one of the most common causes of monocular visual impairment. Intensive investigation has shown that amblyopes suffer from a range of deficits not only in the primary visual cortex but also the extra-striate visual cortex. However, amblyopic brain processing deficits in large-scale information networks especially in the visual network remain unclear.

**Methods:**

Through resting state functional magnetic resonance imaging (rs-fMRI), we studied the functional connectivity and efficiency of the brain visual processing networks in 18 anisometropic amblyopic patients and 18 healthy controls (HCs).

**Results:**

We found a loss of functional correlation within the higher visual network (HVN) and the visuospatial network (VSN) in amblyopes. Additionally, compared with HCs, amblyopic patients exhibited disruptions in local efficiency in the V3v (third visual cortex, ventral part) and V4 (fourth visual cortex) of the HVN, as well as in the PFt, hIP3 (human intraparietal area 3), and BA7p (Brodmann area 7 posterior) of the VSN. No significant alterations were found in the primary visual network (PVN).

**Conclusion:**

Our results indicate that amblyopia results in an intrinsic decrease of both network functional correlations and local efficiencies in the extra-striate visual networks.

## Introduction

Amblyopia (lazy eye), fundamentally a neurological disorder, is characterized by reduced vision in an otherwise normal eye with the presence of an amblyogenic factor, including early child strabismus (ocular misalignment), anisometropia (difference in refractive error), or ametropia (large symmetric refractive errors) and, more rarely, image deprivation (for review, please see Holmes and Clarke [1]). Extensive neuronal physiological studies have found wide-spread within-cortex neural dysfunctions in amblyopic animal models, including a loss of binocularity in V1 [2], an alteration in the excitatory-inhibitory balance of cortical binocular cells [3], disruption of neuronal receptive-field structures [4], and a degradation of neuronal signals [5]. There is also human imaging evidence that amblyopes have abnormal human middle temporal cortex (hMT) response to pattern motion [6], object-related abnormality in high-order occipitotemporal cortex [7], reduced neural adaptation effects in visual cortices [8] and reduced fidelity of spatial representation to the amblyopic eye’s stimulation [9]. Given the fact that the brain is an extraordinarily complex and highly organized network in which dysfunction can spread easily between linked cortices [10, 11], it is so far unclear how the brain neural network is altered by amblyopia.

The analysis of resting-state functional magnetic resonance imaging (rs-fMRI) provides an effective way to assess the brain’s spontaneous activity and connectivity. Brain regions showing synchronized fluctuations during rs-fMRI form the intrinsic connectivity networks (ICNs), which have been demonstrated to provide the physiological basis for cortical information processing, and to be able to abstract and suspend performance influences combined with various neurological diseases [12]. In recent years, the graph theoretical analysis, which defined a graph as a set of nodes (brain areas) and edges (structural or functional connectivity), provided a powerful tool to examine the topological organization of complex brain networks [13–16]. Through these approaches, human brain networks have become an optimum small-world and economical topology [17], represented in characteristics of high global and local efficiency of parallel information processing at a low connection cost [18]*.* Since then, graph theory analysis has been widely and successfully used to explore the brain network architecture in development and neurological diseases, e.g., maturation [19], aging [20], schizophrenia [21], obsessive-compulsive disorder [22], and so on. However, only few rs-fMRI studies investigated amblyopic intrinsic functional connectivity e.g., Ding, Liu [23] found altered connectivity between the primary visual cortex (V1) with the cerebellum and the inferior parietal lobule; Wang, Li [24] have figured out decreased functional connectivity density in the visual ICNs of amblyopic children; and Mendola, Lam [25] have revealed abnormal retinotopically organized functional connectivity of visual areas in amblyopia. It remains unknown whether and how the local efficiency of the brain network evolves from the amblyopes’ abnormal visual experiences.

Here, we measured rs-fMRI to assess 3 ICNs in the visual information processing in 18 healthy volunteers and 18 anisometropic amblyopes: the higher vision network (HVN), the primary visual network (PVN), and the visuospatial network (VSN). Both the intra- and inter-network functional connectivity, as well as the network local efficiency of the visual ICNs were studied. Our results suggest widespread disturbances of functional connectivity and local efficiency in the extra-striate visual networks in amblyopia.

## Materials and methods

### Participants

A group of adult anisometropic amblyopes (*n* = 18, mean age: 23.7 ± 1.9 years old) and a group of healthy controls (n = 18, mean age: 25.2 ± 1.8 years old) participated. Anisometropia was defined as refraction differing by 1.0 diopters (D) or more for the two eyes; amblyopia was defined as reduced visual acuity (> 0.1 LogMAR) an otherwise normal eye due to abnormal visual experience early in life. A brief summary of participants’ clinical data is provided in Table 1. A comprehensive eye examination was carried out by a clinician (the author LF) at the First Affiliated Hospital of Anhui Medical University. No strabismic or eye-pathological cases were reported in the participating individuals. Healthy controls had normal or corrected to normal visual acuity (≤ 0.1 logMAR) in both eyes. All subjects were naive as to the purpose of the experiment. A written informed consent was obtained from each participant after explanation of the nature and possible consequences of the study. This study complied with the Declaration of Helsinki and was approved by the University of Science and Technology of China’s Research Ethics Committee.

**Table 1:** Clinical characteristics of the amblyopes and healthy controls

	Amblyopes	Healthy controls
N	18	18
Age (years)		
Mean ± SD	23.7 ± 1.9	25.2 ± 1.8
Minimum: median: maximum	20: 24: 27	23: 25: 29
Sex		
Female: male	6: 12	4: 14
Best Corrected visual acuity (logMAR)		
Mean ± SD		
Fellow (dominant) eye	0.01 ± 0.06	0.00 ± 0.03
Amblyopic (nondominant) eye	0.59 ± 0.23	0.02 ± 0.04
Minimum: median: maximum		
Fellow (dominant) eye	−0.08: 0.00: 0.10	−0.08: 0.00: 0.10
Amblyopic (nondominant) eye	0.30: 0.56: 1.00	0.00: 0.00: 0.10

### Image acquisition

The collection of magnetic resonance images was performed using a 3.0 T (Signa HDx; GE Healthcare, Illinois, United States) scanner with an eight-channel head coil. Foam padding and earplugs were used to minimize participants’ head motion and reduce scanner noise. Each scanning session began with an acquisition of high-resolution three-dimensional T1 weighted images using an MP-RAGE sequence (TR = 2300 ms; TE = 2.94 ms; flip angle = 9°; 176 slices; voxel size = 1 × 1 × 1 mm^3^). The resting state functional scans were T2*-weighted, gradient-echo, planar images (TR = 2000 ms; TE = 28 ms; flip angle = 72°; 40 slices; voxel size = 3 × 3 × 3 mm^3^). While acquiring resting state images, patients were instructed to keep both eyes closed and to think of nothing in particular.

### MRI data analysis

For fMRI data, preprocessing was applied by using Analysis of Functional NeuroImages (AFNI) software tools (Medical College of Wisconsin, Milwaukee, WI, USA; Cox [26]). The first five images of each resting state session were discarded. Each subject’s fMRI data were registered first to his/her anatomical raw data by linear and quadratic registration. This was followed by slice-timing correction, head motion correction, spatial Gaussian smoothing with a kernel width of 6 mm at half maximum (FWHM), as well as temporal detrending respect to the head motion and order 3 polynomial drift correction. Then, all the images were normalized to the Montreal Neurological Institute (MNI) ICBM152 and were resliced by 3.0 × 3.0 × 3.0 mm^3^ voxels. The motion was assessed and the time points with framewise displacement over 0.2 mm were censored. Data was further filtered with temporal band pass 0.01~0.10 Hz. A regression of motion parameters and their derivatives were applied, and the residual error time series were obtained for further analysis.

**Fig. 1:**
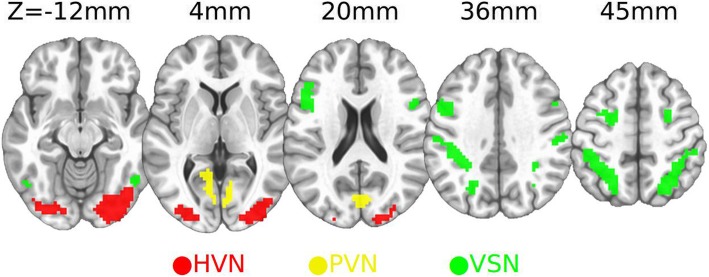
An illustration of the 19 ICN ROIs from the three ICNs (HVN, PVN, and VSN) employed in our study

Our network nodes were constructed by 19 Regions of interest (ROIs) (Fig. 1, Richiardi, Altmann [27]), part of the Willard 499 ROIs, constituting the primary visual network (PVN), higher visual network (HVN), and visuospatial network (VSN); these ROIs were asymmetrically distributed across the brain. ROI labels were in line with brain anatomy by matching the ROI center coordinates to the AFNI Anatomy Toolbox. Functional connectivity between ROIs were calculated using multivariate distance correlation [28]: For example, suppose areas *A* and *B* had *t* time-points, and *v*_*A*_ and *v*_*B*_ voxels, respectively. First, z-transfer was applied to each voxel’s time course by its mean and variance; then, the Euclidean distance, *d*_*A:t1,t2*_ and *d*_*B:t1,t2*_, between each pair of time points *t1* and *t2* was computed for each region:

1 $$ {d}_{A:t1,t2}=\sqrt{\sum_{v=1}^{v_A}{\left({A}_{v,t1}-{\mathrm{A}}_{v,t2}\right)}^2}\kern0.90em \forall t1,t2=1,\dots, t $$

2 $$ {d}_{B:t1,t2}=\sqrt{\sum_{v=1}^B{\left({B}_{v,t1}-{B}_{v,t2}\right)}^2}\kern0.90em \forall t1,t2=1,\dots, t $$

U-centering was applied to set row and column means to zero.

3 $$ {D}_{A:t1,t2}=\left\{\begin{array}{c}{d}_{A:t1,t2}-\frac{1}{t-2}{\sum}_{p=1}^t{d}_{A:t1,p}-\frac{1}{t-2}{\sum}_{q=1}^t{d}_{A:q,t2}+\frac{1}{\left(t-1\right)\left(t-2\right)}{\sum}_{p,q=1}^t{d}_{A:q,p},\mathrm{t}1\ne t2\\ {}0,t1=t2\end{array}\right. $$

The distance correlation, *dCor*, was then computed as follows:

4 $$ dCor\left(A,B\right)=\left\{\begin{array}{c}\sqrt{dCov\left(A,B\right)/\sqrt{dVar(A) dVar(B)}}, dCov\left(A,B\right)>0\\ {}0, dCov\left(A,B\right)\le 0\end{array}\right. $$

where *dCov* was distance covariance and *dVar* was distance variance.

5 $$ dCov\left(A,B\right)=1/t\left(t-3\right){\sum}_{t1,t2=1}^t{D}_{A:t1,t2}{D}_{B:t1,t2} $$

6 $$ dVar(A)=1/t\left(t-3\right){\sum}_{t1,t2=1}^t{D}_{A:t1,t2}^2 $$

The method is similar to the well-established univariate functional connectivity analysis [28], but allows inference based on multivoxel information within each ROI rather than the averaged global BOLD time series. The distance correlation, a metric of multivariate dependence of high dimensional vectors [29], is more reliable and robust than univariate methods [30, 31].

Network edges were obtained by Fisher-transformed distance correlation (*z = 0.5ln[(1 + dCor)/(1- dCor)*], where *dCor* is the distance correlation between the time series of each ROI*,* resulting in a 19*19 functional correlation matrix for each subject. These matrices were further used for the network analysis and for graph theoretical analysis using the GRETNA toolbox [32]. The nodal local efficiency is defined as the harmonic mean of the inverse of the *l*, which is the minimum value of the sum of weights over all possible paths between the immediate neighborhood nodes of the node:

7 $$ {E}_{local}=\frac{1}{N_{G_i}\left({N}_{G_i}-1\right)}{\sum}_{j,k\in {G}_i}\frac{1}{l_{j,k}} $$

where the subgraph *G*_*i*_ is defined as the set of nodes that are directly connected by a single edge to the *i*th node, and *N*_*Gi*_ is the number of nodes in the *G*_*i*_ [18].

The functional connectivity analysis was conducted by the programs in the MATLAB (MathWorks, Natick, MA). The correlation between network node and other node within one network (HVN, PVN, or VSN) is defined as intra-network connectivity, whereas the correlation between a node of a given network and that of another network is defined as the inter-network connectivity. Fisher transformation was applied to intra-and inter-network matrices of each subject to yield mean Fisher-transformed correlation values. The group differences in average intra-network or inter-network were assessed by the repeated-measured analysis of variance (ANOVA), and the false-discovery-rate (FDR) corrected t-test, with *p*-value corrected according to the Algorithm 2 by Storey [33]. Linear correlation analysis were also applied to assess the relationship between the visual acuity and the network connectivity of the amblyopes.

To characterize network efficiency, the local efficiency (LE) of each visual ICN node was computed as a function of the minimum path length between regions [18]. A series of sparsity threshold (0.2 ≤ sparsity ≤ 0.8, interval = 0.05) were applied to measure the individual correlation matrices, for there was no gold standard for selecting a proper single sparsity threshold. The LE at each sparsity was calculated and the area under the curve (AUC) for LE was employed to be a summarized scalar [14]. Group differences in AUC of LE (aLE) of each network node were reported after FDR-corrected t-test ([FDR-corrected] *P* < 0.05) separately.

## Results

### Functional connectivity analysis

**Fig. 2:**
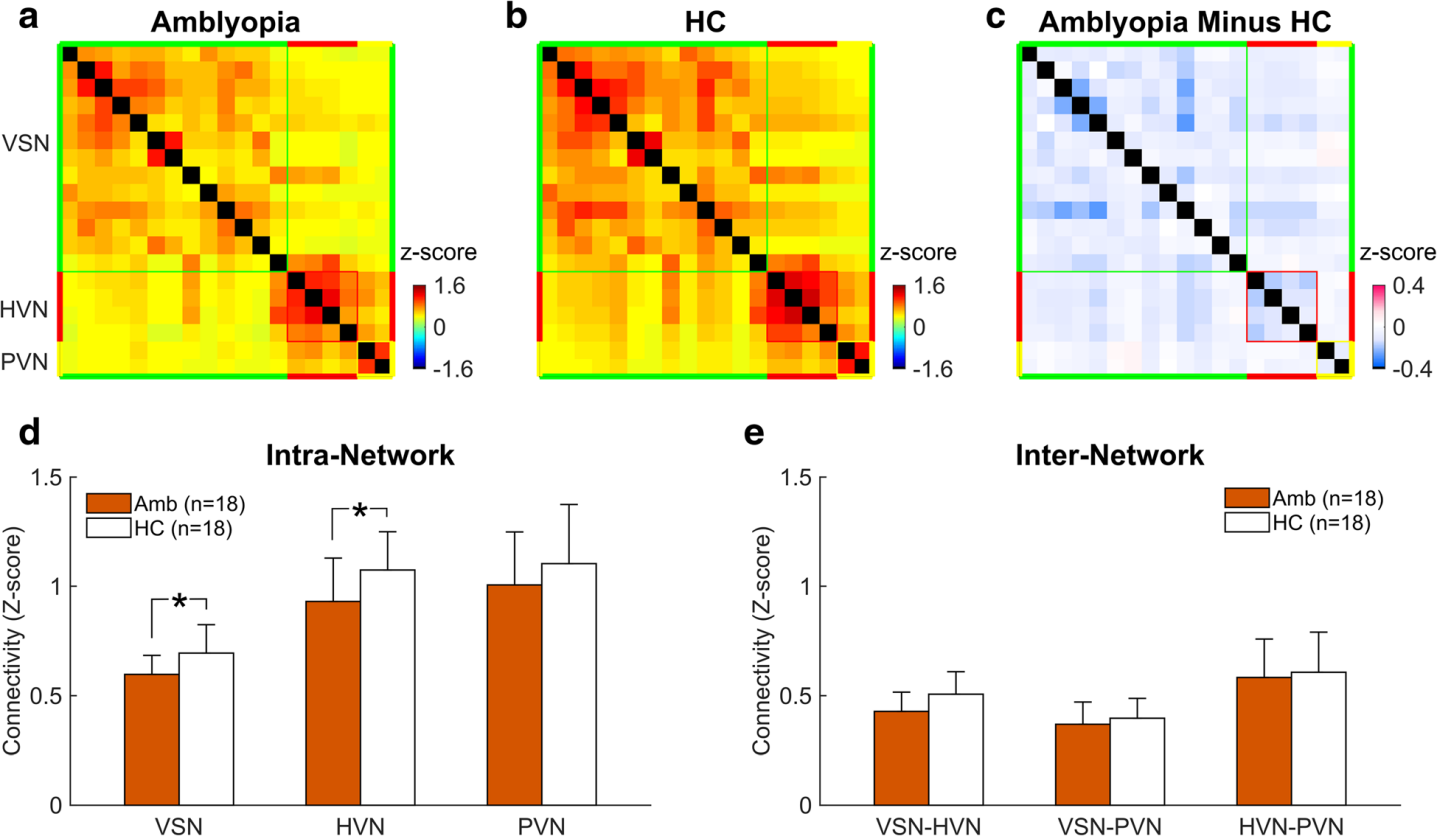
ICN nodes connectivity and group differences**.** 19–19 matrices were computed in all ROIs for all ICNs in amblyopes (**a**) and healthy controls (**b**). The nodes are grouped by ICNs. The intra-network connectivity is plotted as diagonal colored blocks and the inter-network connectivity is plotted as off-diagonal blocks. The group differences are plotted with 19–19 matrices (**c**) and bar graphs (**d**/**e**). Error bars represent standard deviations; *: *P* < 0.05, FDR corrected

Figure 2 shows the results of the functional connectivity analysis for amblyopes (Fig. 2a) and healthy controls (HC; Fig. 2b). As expected, both the HC and the amblyopic matrices showed more positive correlations within each network than those between networks. The amblyopic matrix (Fig. 2a) showed generally reduced correlations compared with the HC matrix (Fig. 2b). This difference was apparent in correlation difference matrix (Amblyopia minus HC) shown by Fig. 2c.

For the intra-network connectivity, we used a mixed-design repeated-measured ANOVA, with group (amblyopes vs. HCs) as the between-subject factor and with intra-network connectivity (HVN, PVN, and VSN) as the within-subject factor. ICN nodes connectivity was significantly different between these two groups (F (1,34) = 4.21, *P* = 0.048); such group difference existed in all the three intra-network conditions, as the interaction between the group and the intra-network was not significant (F (2,68) = 0.15, *P =* 0.86). Two-sample t-test of each ICN further showed that the connectivity within the VSN (t (34) = − 2.64, Uncorrected [FDR corrected] *P =* 0.0124 [0.037]) and the HVN (t (34) = − 2.29, Uncorrected [FDR corrected] *P =* 0.0284 [0.043]) were significantly decreased in amblyopes (Fig. 2d).

Secondly, we performed a mixed-design repeated-measured ANOVA, with group (amblyopes vs. HCs) as the between-subject factor and with inter-network connectivity (HVN-PVN, HVN-VSN, and PVN-VSN) as the within-subject factor. Connectivity was significantly different across the inter-network pairs (F (2,68) = 31.2, *P <* 0.001), while neither the between-group effect (F (1,34) = 1.17, *P =* 0.29) nor the interaction effect (F (2,68) = 1.21, *P =* 0.31) was significant. No significant alteration of between network connectivity was observed in amblyopes (Fig. 2e) after the FDR corrected t-test. We then applied Pearson’s correlation analysis and found that neither intra-network connectivity nor inter-network connectivity showed any significant correlation with the corrected visual acuity in the amblyopes (*P* > 0.5).

### The local efficiency analysis

**Fig. 3:**
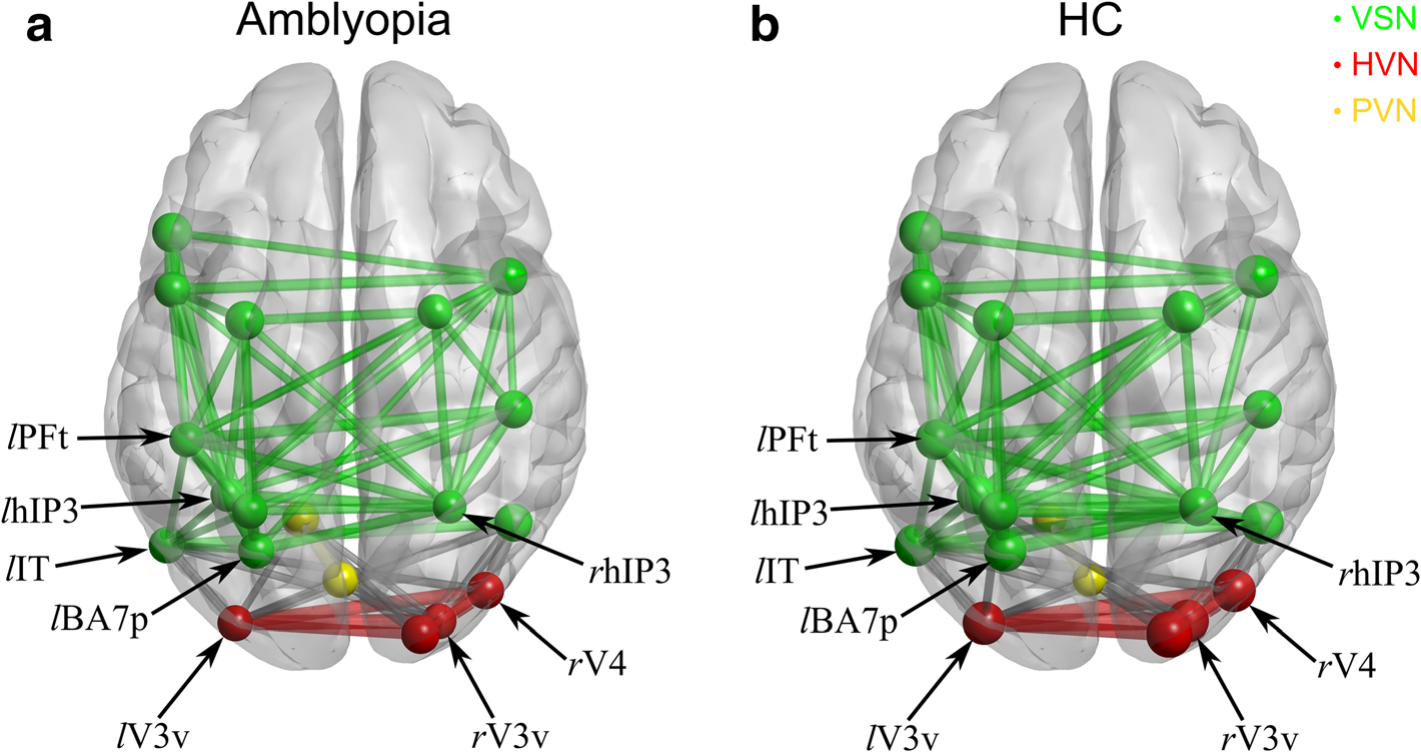
A widespread of extra-striate cortices showed significant decrease of aLE in the amblyopia. The LE was calculated at a series of sparsity threshold (0.2 ≤ sparsity ≤ 0.8, interval = 0.05), and the area under the curve (AUC) for LE (aLE) was obtained as a summarized scalar. FDR-corrected t-test showed a significant decrease of aLE in lPFt, lhIP3, lBA7p, lV3v, rhIP3, rV3v, and rV4 (labelled with arrows) of the amblyopia (smaller node sizes) (**a**) and healthy controls (**b**). Note that a sparsity of 0.35 was used here for illustration, with the sizes of the nodes proportional to the aLE of each node and ICNs labeled with different colors

To further investigate the effects of amblyopia within the visual ICNs, we conducted a local efficiency analysis. In Fig. 3, we plotted the averaged visual ICNs of amblyopes (Fig. 3a) and the HCs (Fig. 3b). The visual ICNs of both groups demonstrated small-world network architecture and the local efficiencies at the extra-striate cortices were significantly decreased in amblyopes, evidenced by the smaller node size illustrated in Fig. 3. A mixed ANOVA, with group (amblyopes vs. HCs) as the between-subject factor and with network nodes as the within-subject factor also showed that there was a significant difference between groups (F (1,34) = 6.27, *P =* 0.017) and nodes (F (18,612) = 2.6, *P* < 0.001). We further conducted a series of t-tests (amblyopes vs. healthy controls) across all the 19 visual ICN nodes. The t-test analysis showed that the aLE at the lPFt, lhIP3, lBA7p, rhIP3, lV3v, rV3v, and rV4 were significantly smaller in amblyopes than that in healthy controls (Table 2).

**Table 2:** MNI coordinates, cortical regions, and the effect of amblyopia on the aLE of each node

ICN	MNI	Cortex	Amblyopia	t-test	HCs
				(*P =* Uncorrected [FDR corrected])	
VSN	−27,-6,55	lFEF	0.402	t = −0.57, *P =* 0.5723 [0.619]	0.423
**VSN**	**−45,-39,47**	lPFt*	**0.361**	**t = −2.64,** ***P =*** **0.0125 [0.034]**	**0.414**
**VSN**	**−33,-55,46**	lhIP3*	**0.353**	**t = −2.66,** ***P =*** **0.0118 [0.034]**	**0.406**
**VSN**	**−24,-73,45**	lBA7p*	**0.372**	**t = − 2.73,** ***P =*** **0.0099 [0.034]**	**0.434**
VSN	− 27,-61,56	lBA7a	0.368	t = − 1.26, *P =* 0.2148 [0.292]	0.398
VSN	−48,5,33	lBA44	0.368	t = − 2.11, *P =* 0.0420 [0.073]	0.419
VSN	− 48,21,21	lBA45	0.408	t = − 1.01, *P =* 0.3196 [0.405]	0.446
VSN	−52,-68,-11	lIT	0.376	t = − 2.29, *P =* 0.0284 [0.067]	0.437
VSN	27,-3,59	rFEF	0.361	t = − 2.12, *P =* 0.0411 [0.073]	0.438
**VSN**	**30,-61,49**	rhIP3*	**0.348**	**t = − 2.78,** ***P =*** **0.0087 [0.034]**	**0.400**
VSN	48,−30,44	rPFt	0.390	t = −0.19, *P =* 0.8509 [0.851]	0.396
VSN	48,8,30	rBA44	0.395	t = −1.61, *P =* 0.1167 [0.171]	0.443
VSN	48,-61,-18	rIT	0.392	t = −1.98, *P =* 0.0562 [0.089]	0.442
**HVN**	**-30,-90,−12**	lV3v*	**0.365**	**t = −2.69,** ***P =*** **0.0110 [0.034]**	**0.438**
**HVN**	**27,-92,-16**	rV3v*	**0.363**	**t = −3.22,** ***P =*** **0.0028 [0.034]**	**0.436**
**HVN**	**42,-83,-16**	rV4*	**0.383**	**t = −2.76,** ***P =*** **0.0093 [0.034]**	**0.441**
HVN	21,-97,11	rV2	0.393	t = −2.13, *P =* 0.0408 [0.073]	0.471
PVN	0,-81,6	rV1	0.368	t = 0.68, *P =* 0.5025 [0.597]	0.343
PVN	-12,-62,-0	lV1	0.381	t = 0.55, *P =* 0.5866 [0.619]	0.354

(bold * indicates significance, *P* < 0.05, FDR corrected)

*MNI* Montreal Neurological Institute, *aLE* area under the curve for local efficiency, *ICN* intrinsic connectivity networks, *HCs* healthy controls, *VSN* visuospatial network, *HVN* higher visual network, *FDR* false discovery rate

## Discussion

Our principal finding is that anisometropic amblyopes suffer from a decrease of intra-network functional connectivity and local efficiency within the brain extra-striate cortices. To our limited knowledge, this study is the first demonstration of an intrinsic alteration of the brain extra-striate visual networks in adult amblyopes, which suggests an underlying pathological process engaged in amblyopia.

Our analysis included distance correlation to assess functional connectivity [29]. This multivariate method was similar to the standard univariate functional connectivity method in obtaining correlations between brain ROIs from resting state fMRI data [30]. Furthermore, the distance correlation, by using multivariate patterns to measure the dependences between two brain regions, could effectively detect the non-linearity while avoiding any within ROI signal averaging. The method is capable of encoding information of associations between brain areas that was lost by averaging [31, 34]. We have also employed a pre-computed group-level brain network parcellation, which represents the functional organization of the brain, and is integrally correlated with genes linked to synaptic function [27].

An important step in understanding how the abnormal visual experience of amblyopia influenced visual neural network is the generation of a map of the connectivity architecture of the brain. The application of network science and graph theory has enabled detailed descriptions of how disease affects the brain [12–16]*.* Through diffusion tensor imaging (DTI), amblyopic brain structural connectivity studies have found increased mean diffusivity (MD) in thalamo-cortical visual pathways [35] and vertical occipital fasciculus [36], as well as decreased fractional anisotropy (FA) in the optic radiation, inferior longitudinal fasciculus/inferior fronto-occipital fasciculus and superior longitudinal fasciculus [37]. Previous rs-fMRI works have reported altered connectivity between the primary visual cortex (V1) with the cerebellum and the inferior parietal lobule [23], decreased functional connectivity density in the visual ICNs in amblyopic children [24], as well as disrupted retinotopically functional connectivity of visual areas in amblyopes [25]. Through network analysis, our present work further delineated the amblyopic deficits in visual network architectures. The observed reduction in the present work suggests that amblyopes have a less efficient visual network compared with that of healthy controls.

A previous study has observed impaired visual functional connectivity in amblyopia while processing the visual information from the amblyopic eye [38]. By using rs-fMRI, we were able to extend the observation to the intrinsic functional connectivity, i.e., no visual inputs. We demonstrated reduced intra-network correlations within the HVN. The deleterious effects of amblyopia on HVN could also be localized in terms of reduced local efficiency of V3v and V4. Since the local efficiency shows how efficient the processing is between the immediate neighbors of a node when the node is removed, it reveals the degree of fault tolerance of the system [39]. Thus, the results suggest that the V3v and V4 were intrinsically less fault tolerant in amblyopes and can be interpreted to have a more fragile visual system intolerant to fault or conflicting information inputs [40, 41].

Furthermore, our results suggest a loss of functional connectivity within the VSN of amblyopes, as well as a reduction of local efficiency of the VSN nodes (hIP3, PFt and BA7p). The VSN are cortices that deal with processing of spatial working memory, visually guided action, eye movements and navigation [42]. The hIP3 has been found to be highly structurally and functionally connected to the visual cortex and plays an important role in attentional selection between peripherally presented stimuli [43]*.* The PFt participates in the action observation and imitation network [44], and the BA7p is a key hub of the VSN bridging to the executive network [45]. Wang, Crewther [46] have found that when amblyopes viewed visual motion stimulus through amblyopic eyes, both the activation and the functional connectivity of VSN were weaker compared to that while viewing through their fellow eyes. Through intrinsic functional network analysis, our results suggested that the amblyopic deficits reflected impaired neural synchronizations within the visuospatial network nodes. This is consistent with a recent study of pathological perturbations to widespread white matter fiber tracts in amblyopia [47]. Our results of reorganization of the visuospatial network that is remote from the primary visual cortex suggest functional pathological cascades encompassing large swathes of the visuospatial system in amblyopia. However, the question of how architecture alterations of the visual networks are linked to amblyopic clinical deficits requires further investigation.

## Conclusions

In summary, we compared the visual ICNs of amblyopes with those of normal observers and found decreased intra-network functional connectivity and local efficiency in some brain areas within the visual ICNs. These findings suggest that amblyopes suffer from a reduction of both internal neural functional connectivity and local efficiency within extra-striates and visuospatial networks.

## Data Availability

The code supporting the findings of this study are available from the corresponding author upon request.

## Abbreviations

ANOVA: Analysis of variance
AUC: Area under curve
BA44: Brodmann area 44
BA45: Brodmann area 45
BA7a: Brodmann area 7 anterior
BA7p: Brodmann area 7 posterior
BOLD: Blood oxygenation level dependent
FA: Fractional anisotropy
FDR: False discovery rate
FEF: Front eye field
fMRI: Functional magnetic resonance imaging
HC: Healthy controls
hIP3: Human intraparietal area 3
hMT: Human middle temporal cortex
HVN: Higher visual network
ICN: Intrinsic connectivity network
IT: Inferior temporal cortex
MD: Mean diffusivity
PVN: Primary visual network
rs-fMRI: Resting state functional magnetic resonance imaging
TE: Echo time
TR: Repetition time
V1: Primary visual cortex
V2: Secondary visual cortex
V3v: Third visual cortex, ventral part
V4: Fourth visual cortex
VOF: Vertical occipital fasciculus
VSN: Visuospatial network

## References

[1] Holmes JM, Clarke MP (2006). Amblyopia. Lancet..

[2] Hubel DH, Wiesel TN (1965). Binocular interaction in striate cortex of kittens reared with artificial squint. J Neurophysiol.

[3] Sengpiel F, Blakemore C (1996). The neural basis of suppression and amblyopia in strabismus. Eye..

[4] Tao X, Zhang B, Shen G, Wensveen J, Smith EL, Nishimoto S (2014). Early monocular defocus disrupts the normal development of receptive-field structure in V2 neurons of macaque monkeys. J Neurosci.

[5] Shooner C, Hallum LE, Kumbhani RD, Ziemba CM, Garcia-Marin V, Kelly JG (2015). Population representation of visual information in areas V1 and V2 of amblyopic macaques. Vis Res.

[6] Thompson B, Villeneuve MY, Casanova C, Hess RF (2012). Abnormal cortical processing of pattern motion in amblyopia: evidence from fMRI. NeuroImage..

[7] Lerner Y, Pianka P, Azmon B, Leiba H, Stolovitch C, Loewenstein A (2003). Area-specific amblyopic effects in human occipitotemporal object representations. Neuron..

[8] Li X, Coyle D, Maguire L, McGinnity TM, Hess RF (2011). Long timescale fMRI neuronal adaptation effects in human amblyopic cortex. PLoS One.

[9] Farivar R, Zhou J, Huang Y, Feng L, Zhou Y, Hess RF (2019). Two cortical deficits underlie amblyopia: a multifocal fMRI analysis. NeuroImage..

[10] Tononi G, Sporns O, Edelman GM (1994). A measure for brain complexity: relating functional segregation and integration in the nervous system. Proc Natl Acad Sci U S A.

[11] Fornito A, Zalesky A, Breakspear M (2015). The connectomics of brain disorders. Nat Rev Neurosci.

[12] Ewers M, Sperling RA, Klunk WE, Weiner MW, Hampel H (2011). Neuroimaging markers for the prediction and early diagnosis of Alzheimer's disease dementia. Trends Neurosci.

[13] Bullmore E, Sporns O (2009). Complex brain networks: graph theoretical analysis of structural and functional systems. Nat Rev Neurosci.

[14] He Y, Evans A (2010). Graph theoretical modeling of brain connectivity. Curr Opin Neurol.

[15] Bassett DS, Bullmore E, Verchinski BA, Mattay VS, Weinberger DR, Meyer-Lindenberg A (2008). Hierarchical organization of human cortical networks in health and schizophrenia. J Neurosci.

[16] Salvador R, Suckling J, Coleman MR, Pickard JD, Menon D, Bullmore E (2005). Neurophysiological architecture of functional magnetic resonance images of human brain. Cereb Cortex.

[17] Watts DJ, Strogatz SH (1998). Collective dynamics of 'small-world' networks. Nature..

[18] Achard S, Bullmore E (2007). Efficiency and cost of economical brain functional networks. PLoS Comput Biol.

[19] Zhong S, He Y, Shu H, Gong G (2017). Developmental changes in topological asymmetry between hemispheric brain white matter networks from adolescence to young adulthood. Cereb Cortex.

[20] Cotier FA, Zhang R, Lee TMC (2017). A longitudinal study of the effect of short-term meditation training on functional network organization of the aging brain. Sci Rep.

[21] Zhu J, Wang C, Liu F, Qin W, Li J, Zhuo C (2016). Alterations of functional and structural networks in schizophrenia patients with auditory verbal hallucinations. Front Hum Neurosci.

[22] Jung WH, Yucel M, Yun JY, Yoon YB, Cho KI, Parkes L (2017). Altered functional network architecture in orbitofronto-striato-thalamic circuit of unmedicated patients with obsessive-compulsive disorder. Hum Brain Mapp.

[23] Ding K, Liu Y, Yan X, Lin X, Jiang T (2013). Altered functional connectivity of the primary visual cortex in subjects with amblyopia. Neural Plast.

[24] Wang T, Li Q, Guo M, Peng Y, Li Q, Qin W (2014). Abnormal functional connectivity density in children with anisometropic amblyopia at resting-state. Brain Res.

[25] Mendola JD, Lam J, Rosenstein M, Lewis LB, Shmuel A (2018). Partial correlation analysis reveals abnormal retinotopically organized functional connectivity of visual areas in amblyopia. Neuroimage Clin.

[26] Cox RW (1996). AFNI: software for analysis and visualization of functional magnetic resonance neuroimages. Comput Biomed Res.

[27] Richiardi J, Altmann A, Milazzo AC, Chang C, Chakravarty MM, Banaschewski T (2015). Correlated gene expression supports synchronous activity in brain networks. Science..

[28] Geerligs Linda, Cam-CAN, Henson Richard N. (2016). Functional connectivity and structural covariance between regions of interest can be measured more accurately using multivariate distance correlation. NeuroImage.

[29] Székely GJ, Rizzo ML, Bakirov NK (2007). Measuring and testing dependence by correlation of distances. Ann Stat.

[30] Geerligs Linda, Tsvetanov Kamen A., Cam-CAN, Henson Richard N. (2017). Challenges in measuring individual differences in functional connectivity using fMRI: The case of healthy aging. Human Brain Mapping.

[31] Yoo K, Rosenberg MD, Noble S, Scheinost D, Constable RT, Chun MM (2019). Multivariate approaches improve the reliability and validity of functional connectivity and prediction of individual behaviors. Neuroimage..

[32] Wang J, Wang X, Xia M, Liao X, Evans A, He Y (2015). GRETNA: a graph theoretical network analysis toolbox for imaging connectomics. Front Hum Neurosci.

[33] Storey JD (2002). A direct approach to false discovery rates. J R Stat Soc Ser B (Stat Methodol).

[34] Anzellotti S, Coutanche MN (2018). Beyond functional connectivity: investigating networks of multivariate representations. Trends Cogn Sci.

[35] Allen B, Spiegel DP, Thompson B, Pestilli F, Rokers B (2015). Altered white matter in early visual pathways of humans with amblyopia. Vis Res.

[36] Duan Y, Norcia AM, Yeatman JD, Mezer A (2015). The structural properties of major white matter tracts in strabismic amblyopia. Invest Ophthalmol Vis Sci.

[37] Li Q, Jiang Q, Guo M, Li Q, Cai C, Yin X (2013). Grey and white matter changes in children with monocular amblyopia: voxel-based morphometry and diffusion tensor imaging study. Br J Ophthalmol.

[38] Li X, Mullen KT, Thompson B, Hess RF (2011). Effective connectivity anomalies in human amblyopia. Neuroimage.

[39] Latora V, Marchiori M (2001). Efficient behavior of small-world networks. Phys Rev Lett.

[40] Zhou JW, Liu R, Feng LX, Zhou YF, Hess RF (2016). Deficient binocular combination of second-order stimuli in amblyopia. Invest Ophthalmol Vis Sci.

[41] Huang CB, Zhou J, Lu ZL, Feng L, Zhou Y (2009). Binocular combination in anisometropic amblyopia. J Vision.

[42] Kravitz DJ, Saleem KS, Baker CI, Mishkin M (2011). A new neural framework for visuospatial processing. Nat Rev Neurosci.

[43] Gillebert CR, Mantini D, Peeters R, Dupont P, Vandenberghe R (2013). Cytoarchitectonic mapping of attentional selection and reorienting in parietal cortex. NeuroImage..

[44] Caspers S, Zilles K, Laird AR, Eickhoff SB (2010). ALE meta-analysis of action observation and imitation in the human brain. Neuroimage.

[45] Humphreys GF, Lambon Ralph MA (2015). Fusion and fission of cognitive functions in the human parietal cortex. Cereb Cortex.

[46] Wang H, Crewther SG, Liang M, Laycock R, Yu T, Alexander B (2017). Impaired activation of visual attention network for motion salience is accompanied by reduced functional connectivity between frontal eye fields and visual cortex in strabismic amblyopia. Front Hum Neurosci.

[47] Tsai TH, Su HT, Hsu YC, Shih YC, Chen CC, Hu FR (2019). White matter microstructural alterations in amblyopic adults revealed by diffusion spectrum imaging with systematic tract-based automatic analysis. Br J Ophthalmol.

